# Supplementary material for: Nested Spatial and Temporal Modeling of Environmental Conditions Associated With Genetic Markers of Vibrio parahaemolyticus in Washington State Pacific Oysters
Source: Front Microbiol. 2022 Mar 30;13:849336. doi: 10.3389/fmicb.2022.849336 (PMC9007611; doi:10.3389/fmicb.2022.849336)
Supplement: Supplementary file 2 [file Data_Sheet_2.docx]

Supplementary Material

**Nested spatial and temporal modeling of environmental conditions associated with genetic markers of *Vibrio parahaemolyticus* in Washington state Pacific oysters**

Brendan Fries, Benjamin J. K. Davis, Anne E. Corrigan, Angelo DePaola, and Frank C. Curriero


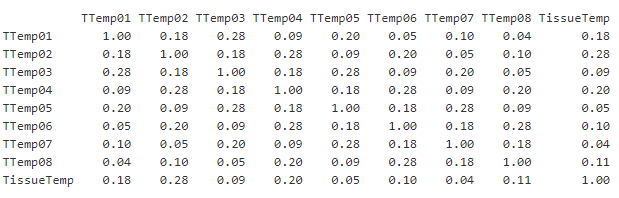
**Table S1.** Correlation matrices for time-indexed compared to lagged variables from 1-8 weeks lagged

Tissue Temperature


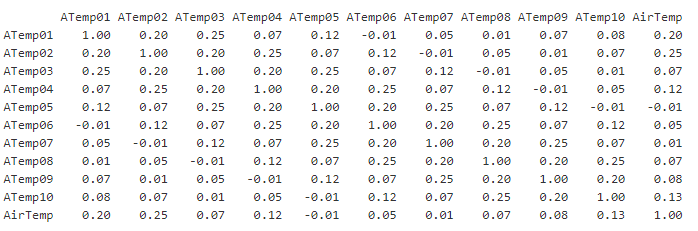

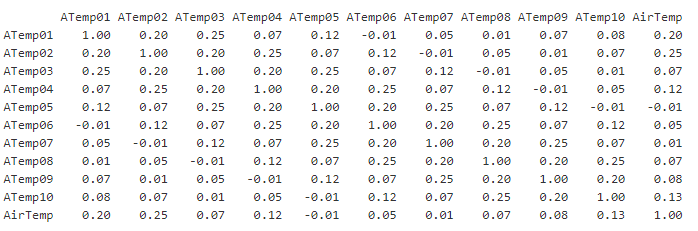
Air Temperature


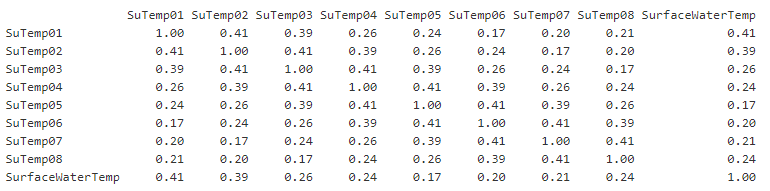
Surface Water Temperature


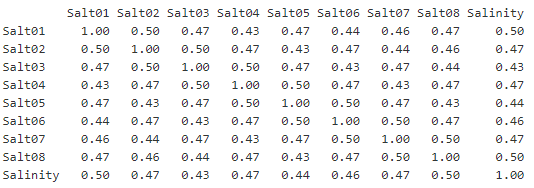
Salinity

| **Table S2.** Univariate and multivariate associations between *trh:tlh* and *tdh:trh* ratios and environmental covariates. | **log *trh:tlh* log *tdh:trh*** | | | | | |  | |  | |  | |
| --- | --- | --- | --- | --- | --- | --- | --- | --- | --- | --- | --- | --- |
|  | **Univariate** | **Multivariate** | **Univariate** | **Multivariate** |  |  | |  | |  | |  |
| **Ecological characteristic** | | | | | |  | |  | |  | |  |
| Salinity (ppt) - 3 week lag | - | - | 0.02 (0.01, 0.04) | 0.01 (0.00, 0.03) |  |  | |  | |  | |  |
| *1 ppt - 27 ppt* | -0.20 (-0.48, 0.07) | -0.12 (-0.40, 0.15) | - | - | - |  | |  | |  | |  |
| *27 ppt - 35 ppt* | 0.39 (0.05, 0.73) | 0.39 (0.06, 0.72) | - | - |  |  | |  | |  | |  |
| Tissue Temp (°C) | 0.00 (0.00, 0.01) | * | -0.03 (-0.04, -0.02) | * | - |  | |  | |  | |  |
| Air Temp (°C) - 1 week lag | -0.01 (-0.02, 0.00) | * | -0.02 (-0.05, -0.02) | * | - |  | |  | |  | |  |
| Surface Water Temp (°C) | -0.02 (-0.03, 0.00) | -0.02 (-0.04, 0.00) | -0.08 (-0.10, -0.06) | -0.06 (-0.09, -0.04) |  |  | |  | |  | |  |
|  |  |  |  |  |  |  | |  | |  | |  |

*Results are displayed as the log-transformed, pooled parameter estimates of the model with associated 95% confidence intervals. Reported associations are adjusted for region and year. * Indicated null effect (0) and exclusion from multivariate model.*

**Table S3.** Model Random Effects for Zone and Sample Site Year Group (SSYG)

|  | **log *trh:tlh* log *tdh:trh*** | | | | | | | | | | |  | | | |
| --- | --- | --- | --- | --- | --- | --- | --- | --- | --- | --- | --- | --- | --- | --- | --- |
| **Random Effects** | | | | | | | | | | |  | | | | |
| **Univariate**  Salinity (ppt) |  | |  |  | | |  | | | | | | |  |  |
| *Random Intercept - Zone* | 0.33 | | 0.01 |  |  |  |  |  |  |  |  |  |  |  |  |
| *Random Intercept - SSYG* | 0.23 | | 0.23 |  |  |  |  |  |  |  |  |  |  |  |  |
| Tissue Temp (°C) |  | |  |  | | |  | | | | | | |  |  |
| *Random Intercept - Zone* | 0.37 | | 0.00 |  |  |  |  |  |  |  |  |  |  |  |  |
| *Random Intercept - SSYG* | 0.25 | | 0.25 |  |  |  |  |  |  |  |  |  |  |  |  |
| Air Temp (°C) |  | |  |  | | |  | | | | | | |  |  |
| *Random Intercept - Zone* | 0.36 | | 0.00 |  |  |  |  |  |  |  |  |  |  |  |  |
| *Random Intercept - SSYG* | 0.25 | | 0.25 |  |  |  |  |  |  |  |  |  |  |  |  |
| Surface Water Temp (°C) |  | |  |  | | |  | | | | | |  |  |  |
| *Random Intercept - Zone* | 0.35 | | 0.00 |  | | | | | |  |  |  |  |  |  |
| *Random Intercept - SSYG* | 0.24 | | 0.25 |  | | | | | |  |  |  |  |  |  |
| **Multivariate** |  | |  |  | |  | |  | | | | | | |  |
| *Random Intercept - Zone* | 0.30 | 0.00 | |  |  |  |  |  |  |  |  |  |  |  |  |
| *Random Intercept - SSYG* | 0.11 | 0.01 | |  |  |  |  |  |  |  |  |  |  |  |  |
| *Estimate of random intercept effect size for univariate and multivariate models.* |  | |  |  |  | | | |  | |  | | | | |
