# Supplementary material for: Nested Spatial and Temporal Modeling of Environmental Conditions Associated With Genetic Markers of Vibrio parahaemolyticus in Washington State Pacific Oysters
Source: Front Microbiol. 2022 Mar 30;13:849336. doi: 10.3389/fmicb.2022.849336 (PMC9007611; doi:10.3389/fmicb.2022.849336)
Supplement: Supplementary file 3 [file Data_Sheet_3.pdf]

Supplementary Material

Nested spatial and temporal modeling of environmental conditions associated with genetic markers of *Vibrio parahaemolyticus* in Washington state Pacific oysters

Brendan Fries, Benjamin J. K. Davis, Anne E. Corrigan, Angelo DePaola, and Frank C. Curriero

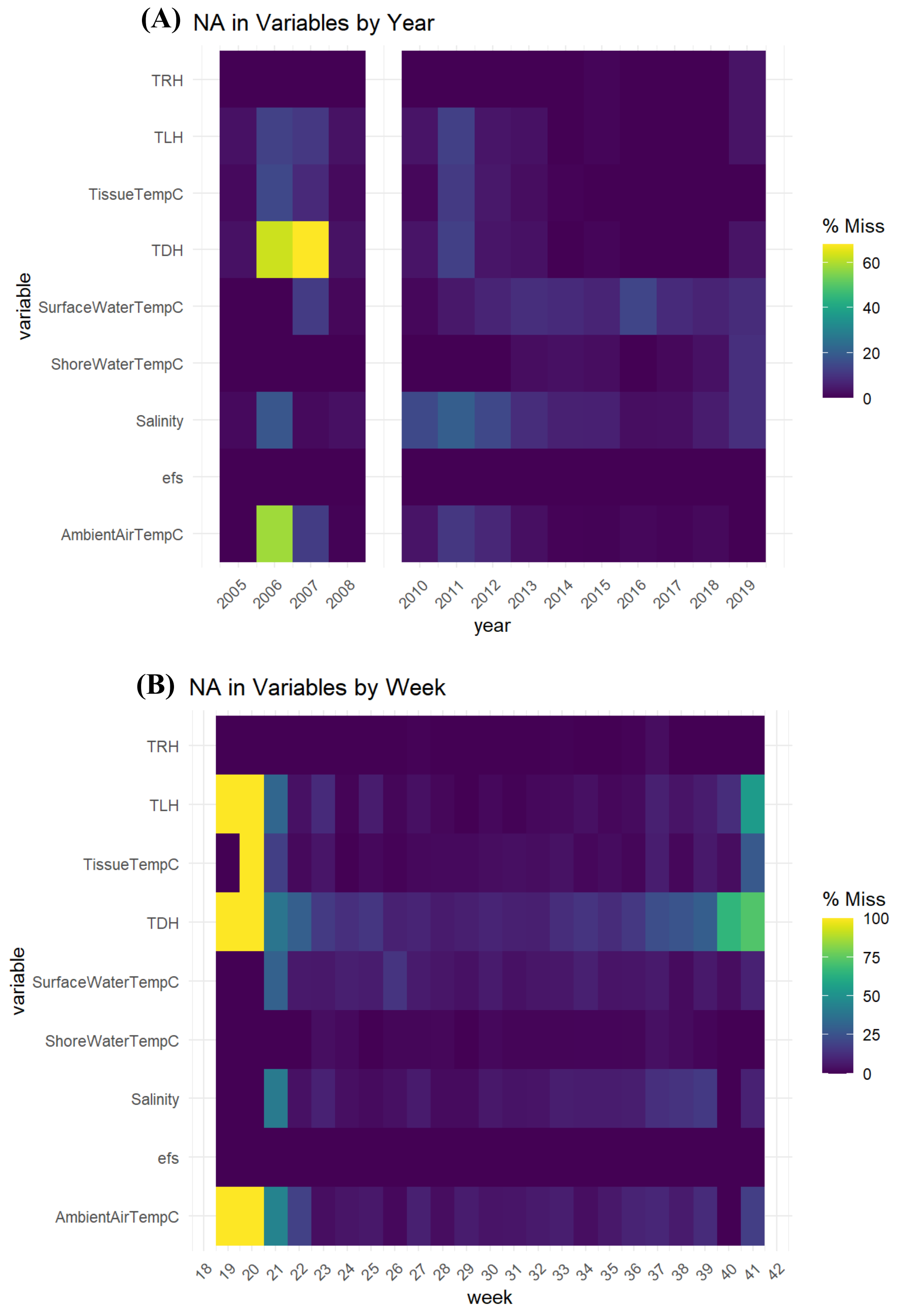

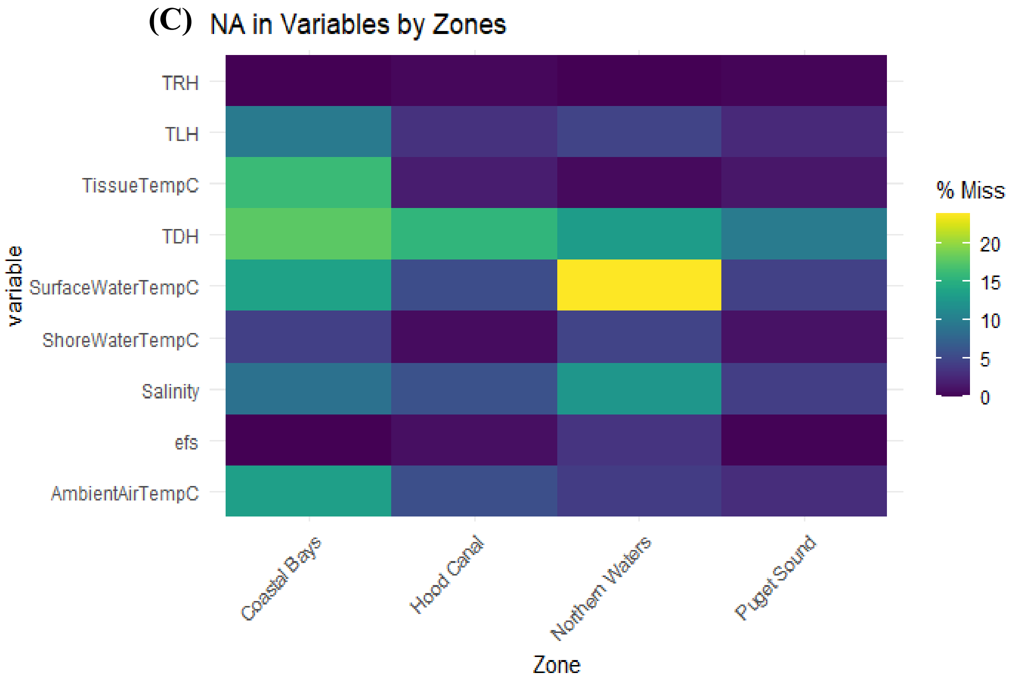

Figure S1. (A): Pre-Imputation Dataset Missingness by year (2009 excluded). (B): Pre-Imputation Dataset Missingness by sampling week in year. (C): Pre-Imputation Dataset Missingness by sampling Zone.

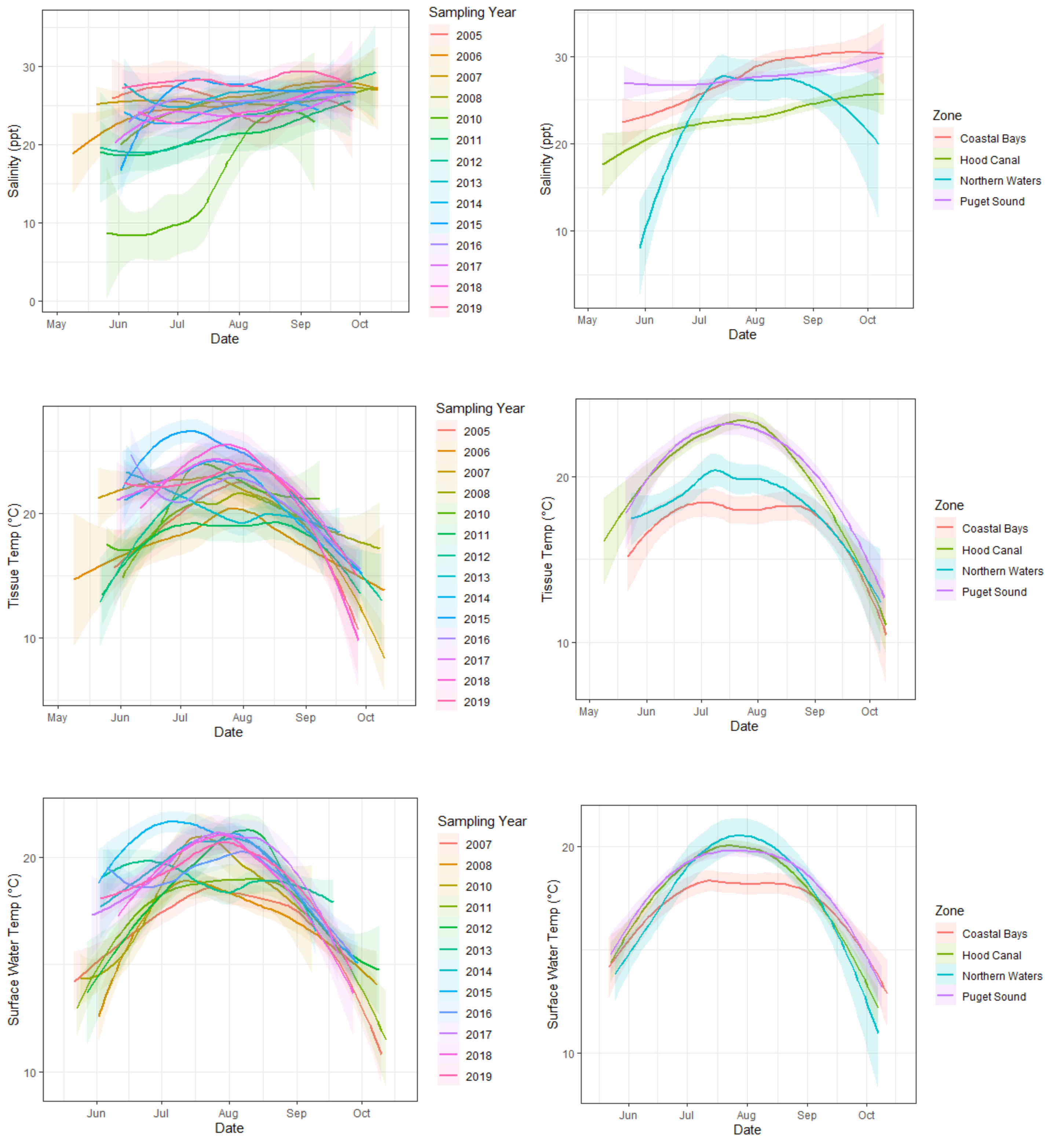

Figure S2. LOESS plots of environmental characteristics over intra-annual time, stratified by year and zone.

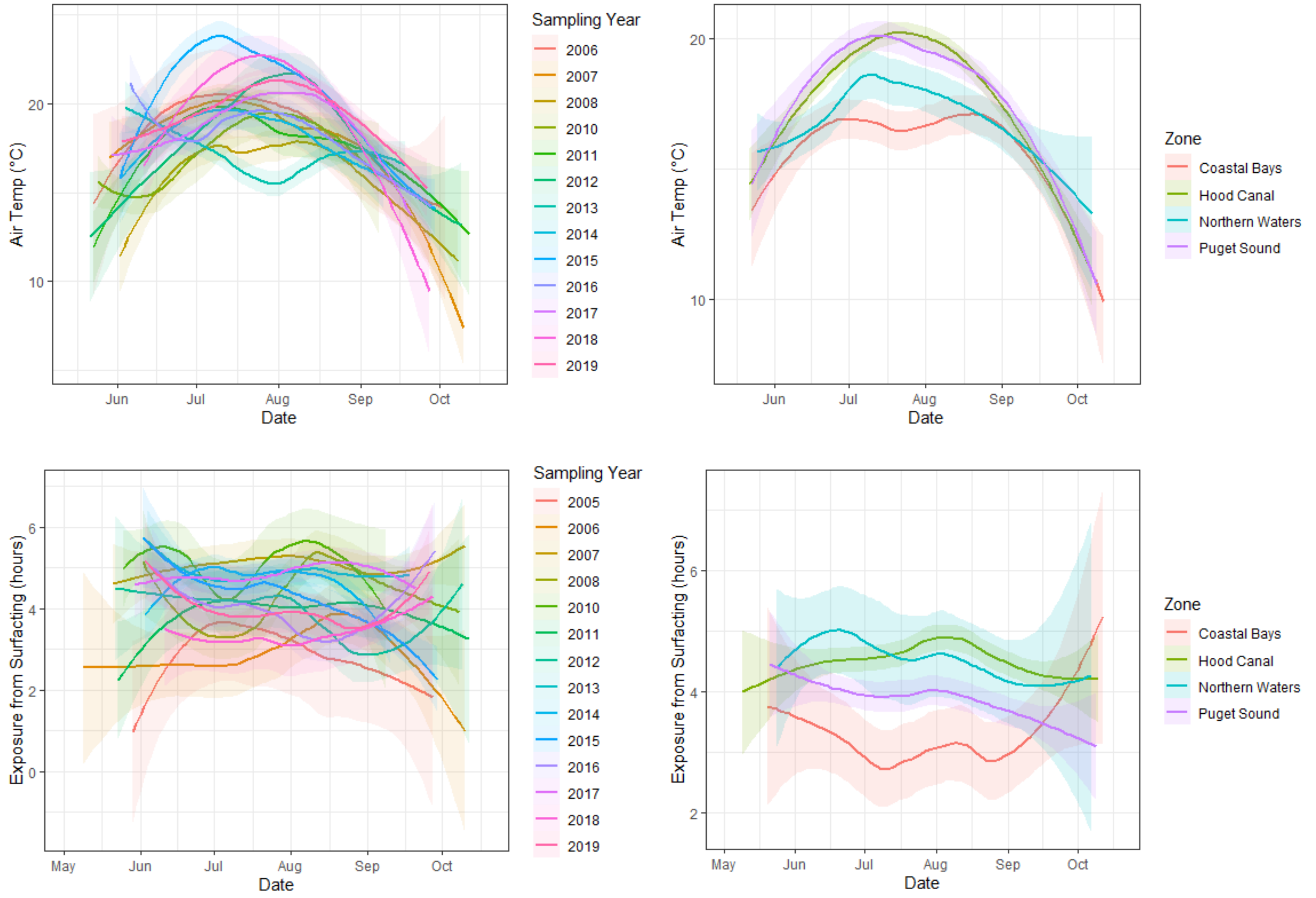

Figure S2 cont. LOESS plots of environmental characteristics over intra-annual time, stratified by year and zone.

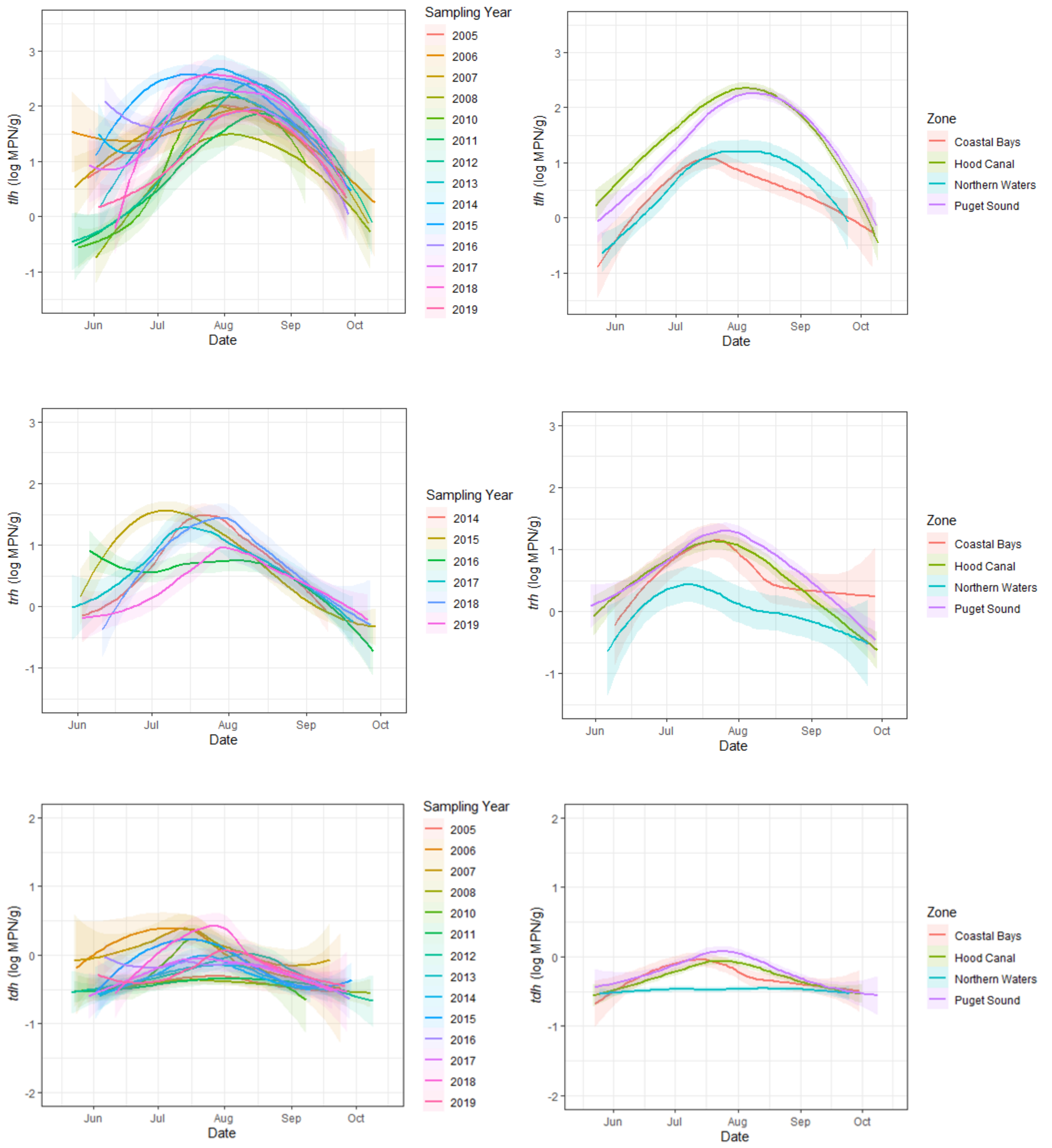

Figure S3. LOESS plots of *V. parahaemolyticus* genetic markers over intra-annual time, stratified by year and zone. Plots display a representative imputation for samples below and above the limit of detection.

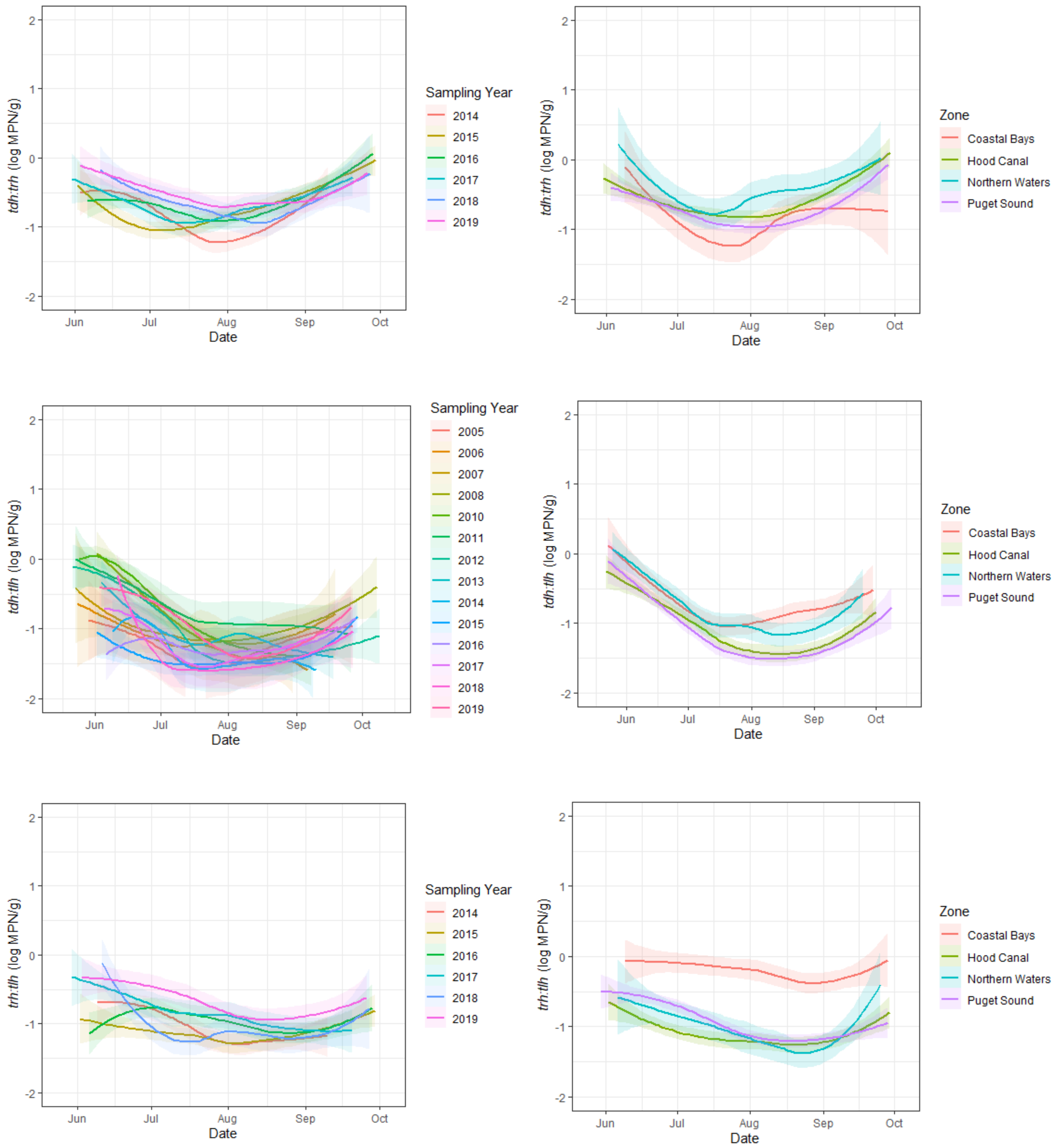

Figure S4. LOESS plots of *V. parahaemolyticus* genetic marker ratios over intra-annual time, stratified by year and zone. Plots display a representative imputation for samples below and above the limit of detection.

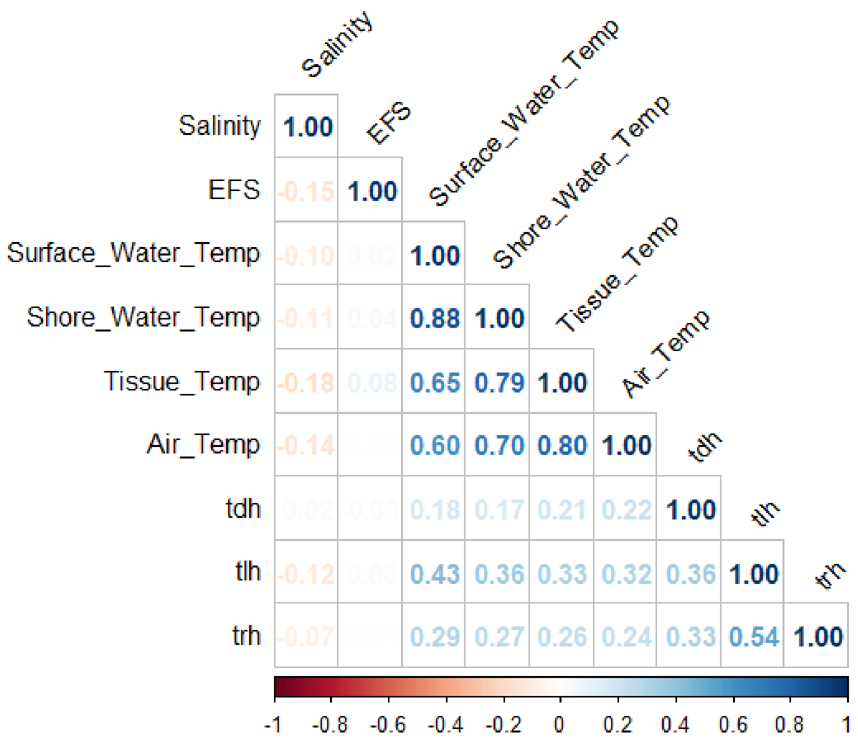

Figure S5. Correlation matrix of covariate values

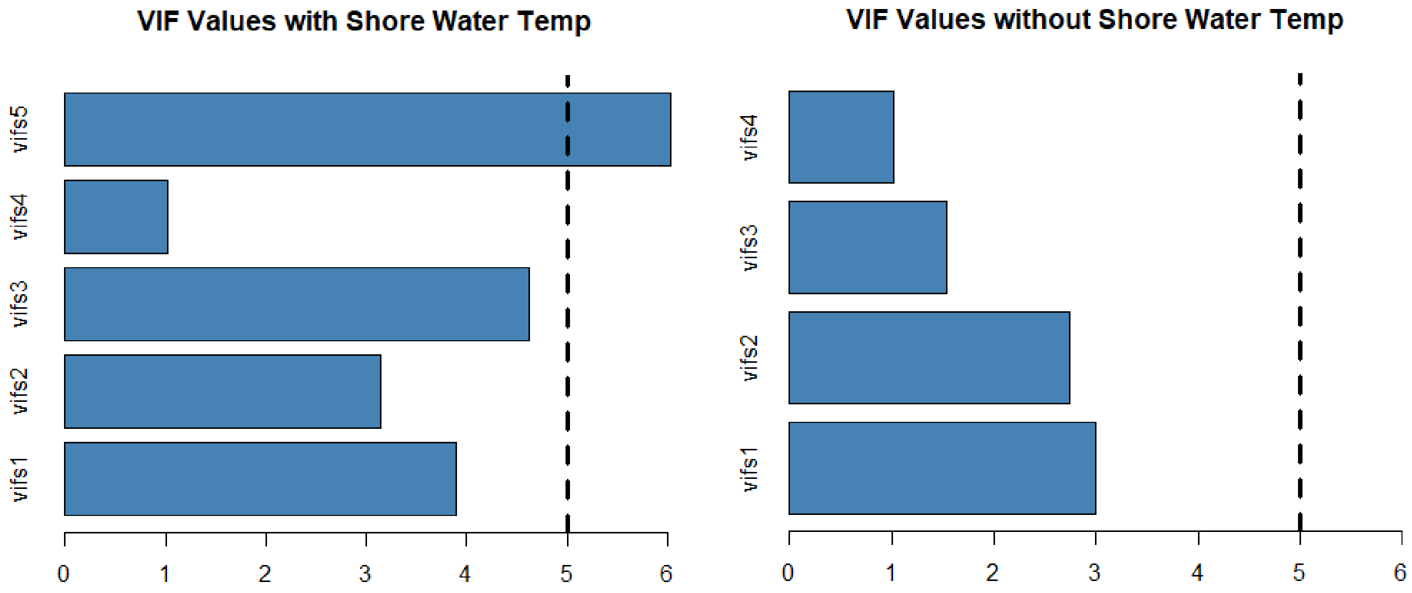

Figure S6. Variance inflation factors of model including and excluding shore water temperature. Variables vifs1 = tissue temperature, vifs2 = Air temperature, vifs3 = surface water temperature, vifs4 = salinity, vifs5 = shore water temperature.

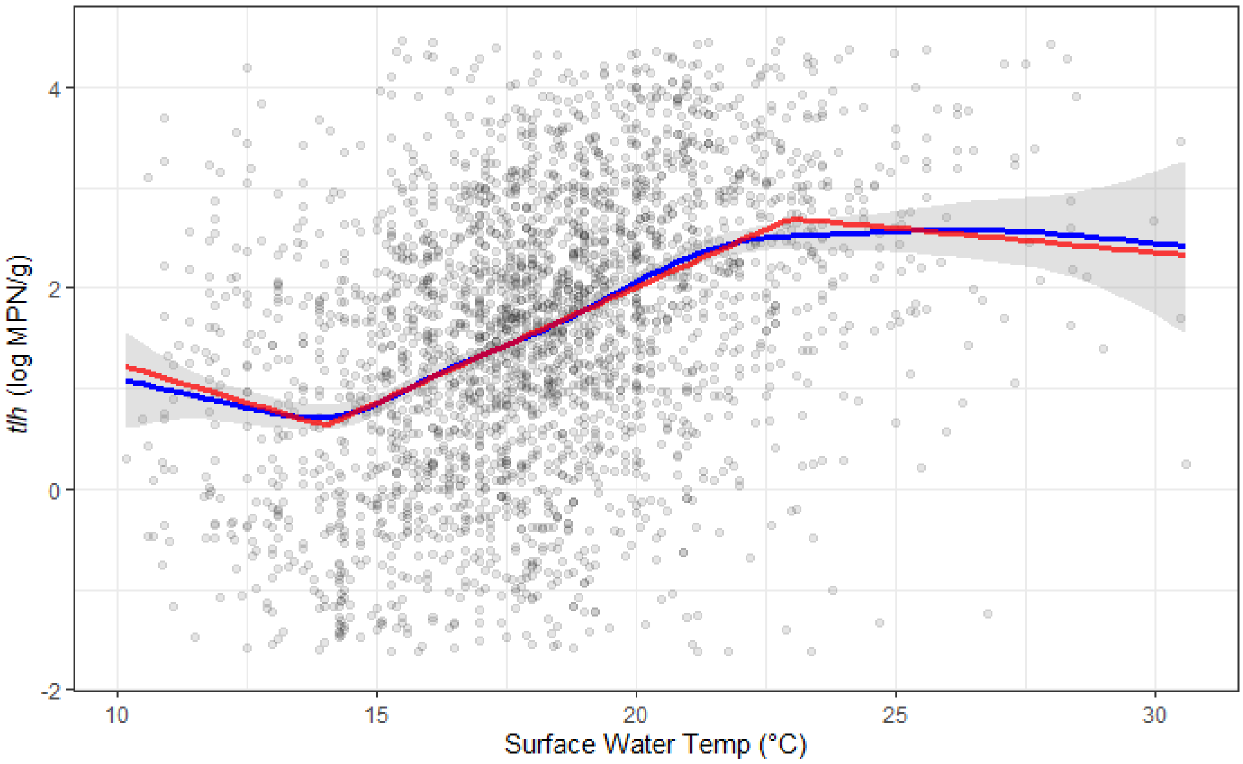

Figure S7A. Comparison of univariate regressions for surface water temperature to *tlh* with linear regression with spline term (red) compared to smoothed conditional mean line of data (blue).

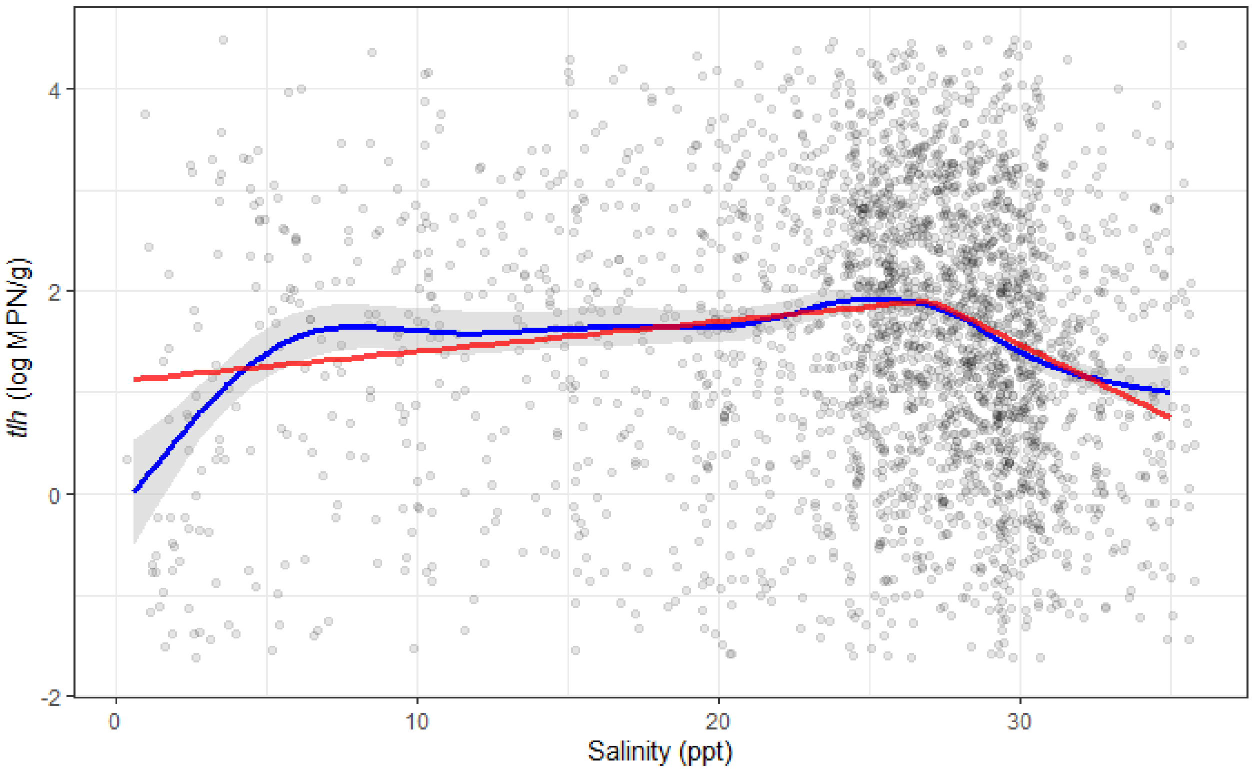

Figure S7B. Comparison of univariate regressions for salinity to *tlh* with linear regression with spline term (red) compared to smoothed conditional mean line of data (blue).

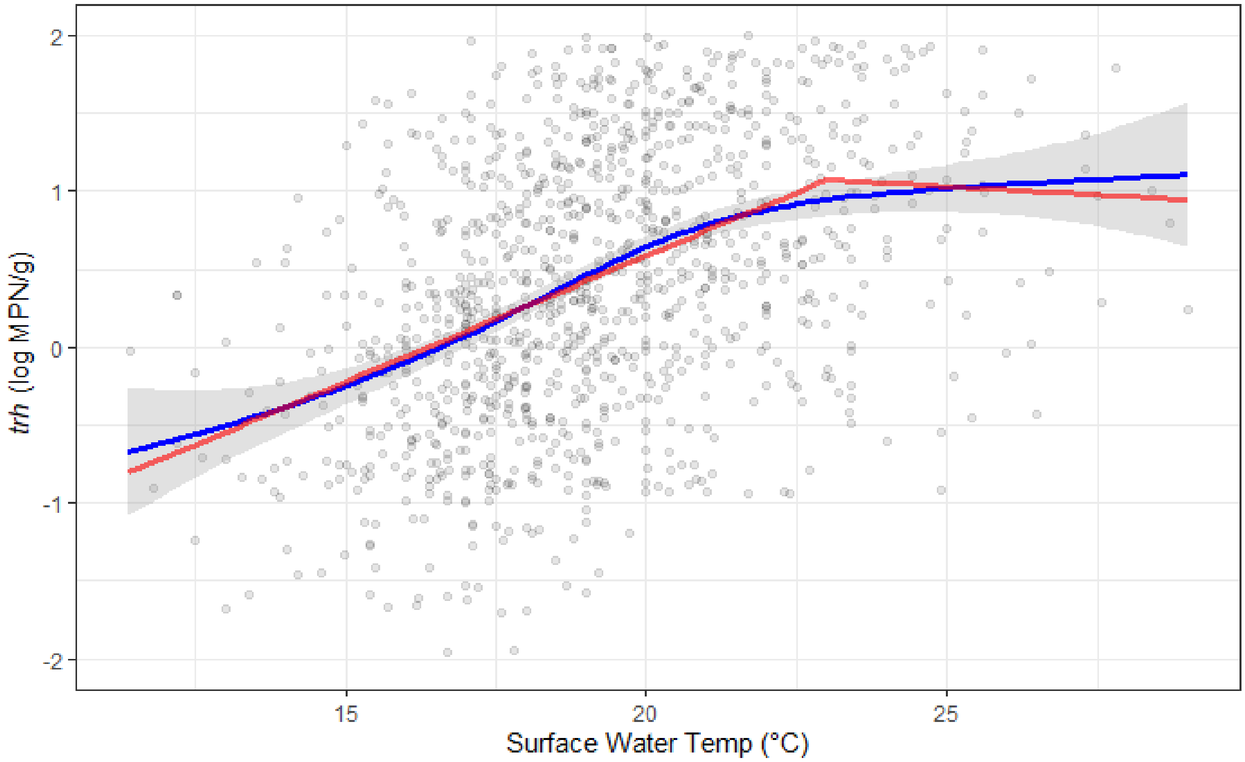

Figure S7C. Comparison of univariate regressions for surface water temperature to *trh* with linear regression with spline term (red) compared to smoothed conditional mean line of data (blue).

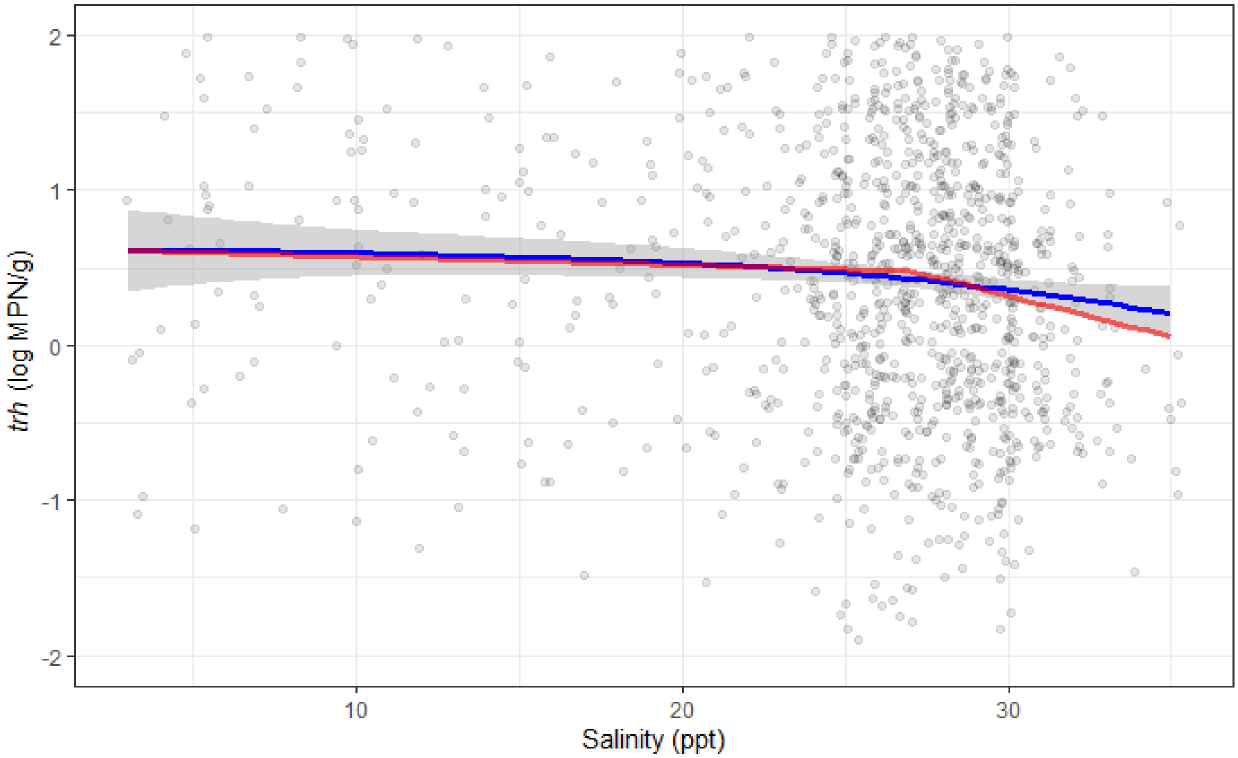

Figure S7D. Comparison of univariate regressions for salinity to *trh* with linear regression with spline term (red) compared to smoothed conditional mean line of data (blue).

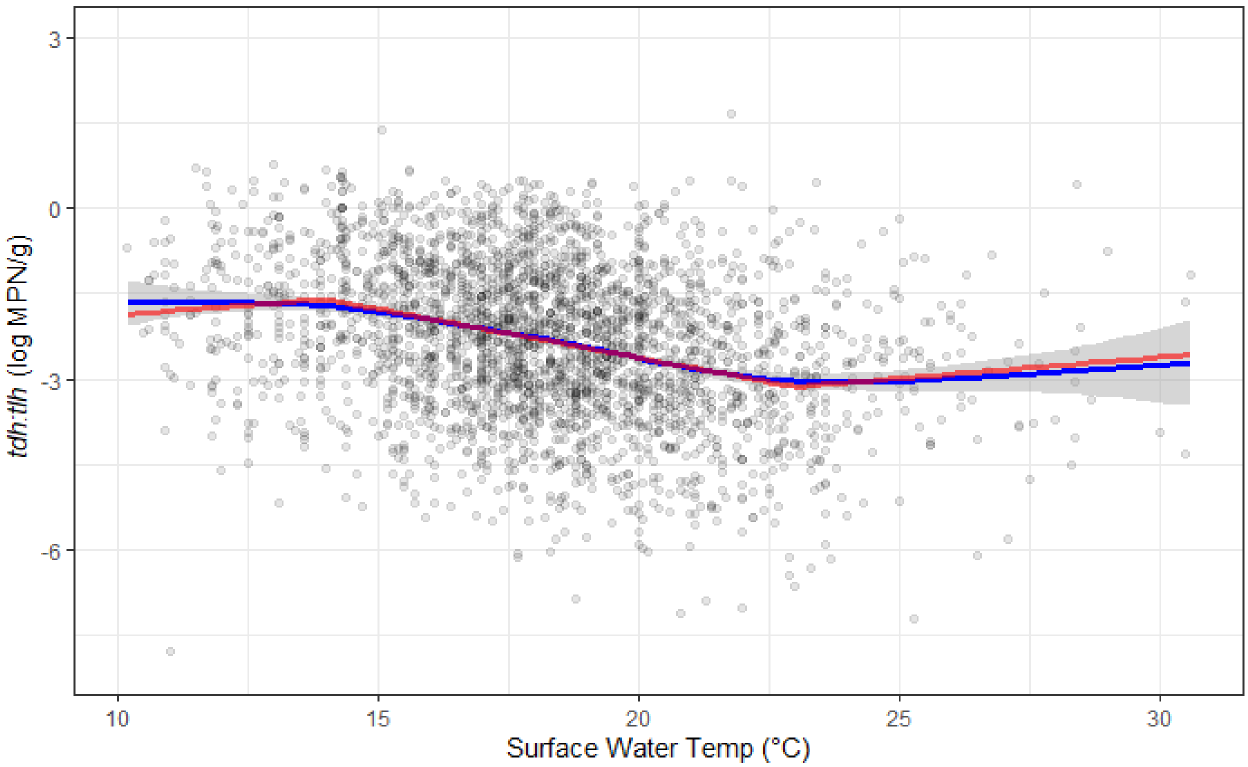

Figure S7E. Comparison of univariate regressions for salinity to *tdh*:*tlh* with linear regression with spline term (red) compared to smoothed conditional mean line of data (blue).

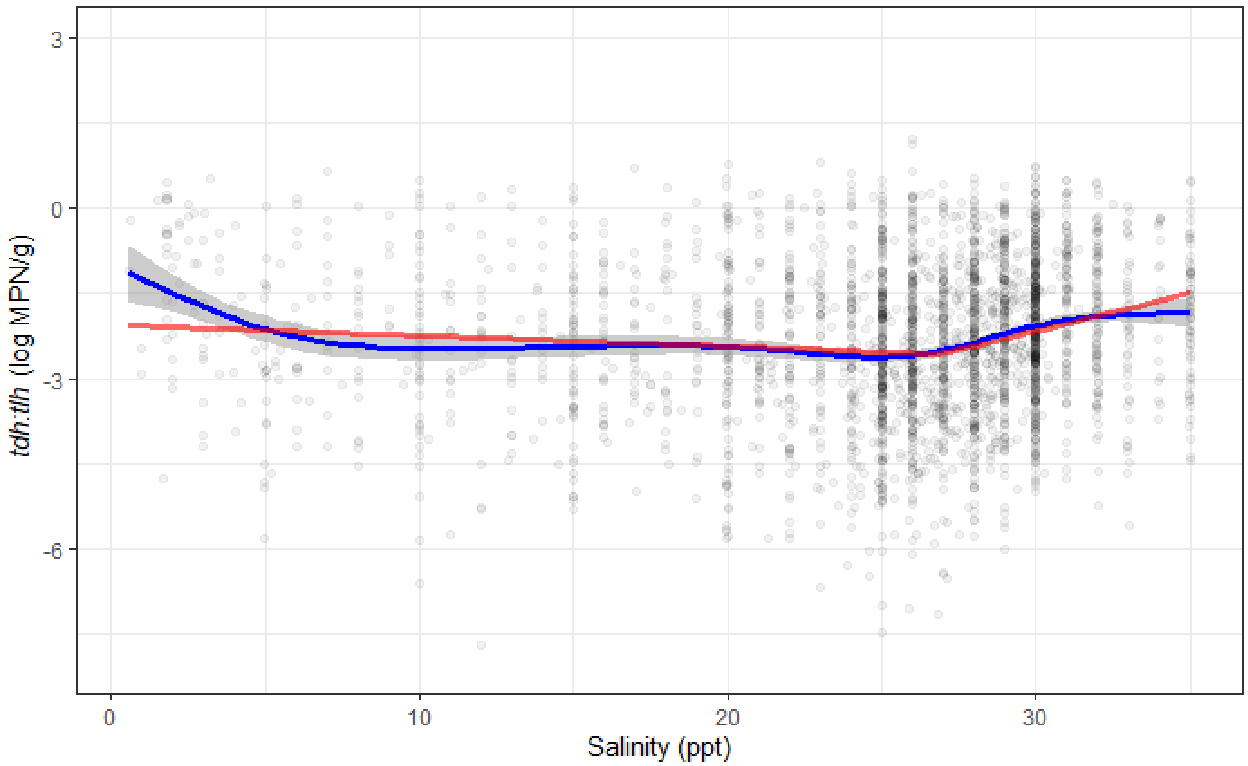

Figure S7F. Comparison of univariate regressions for salinity to *tdh*:*tlh* with linear regression with spline term (red) compared to smoothed conditional mean line of data (blue).

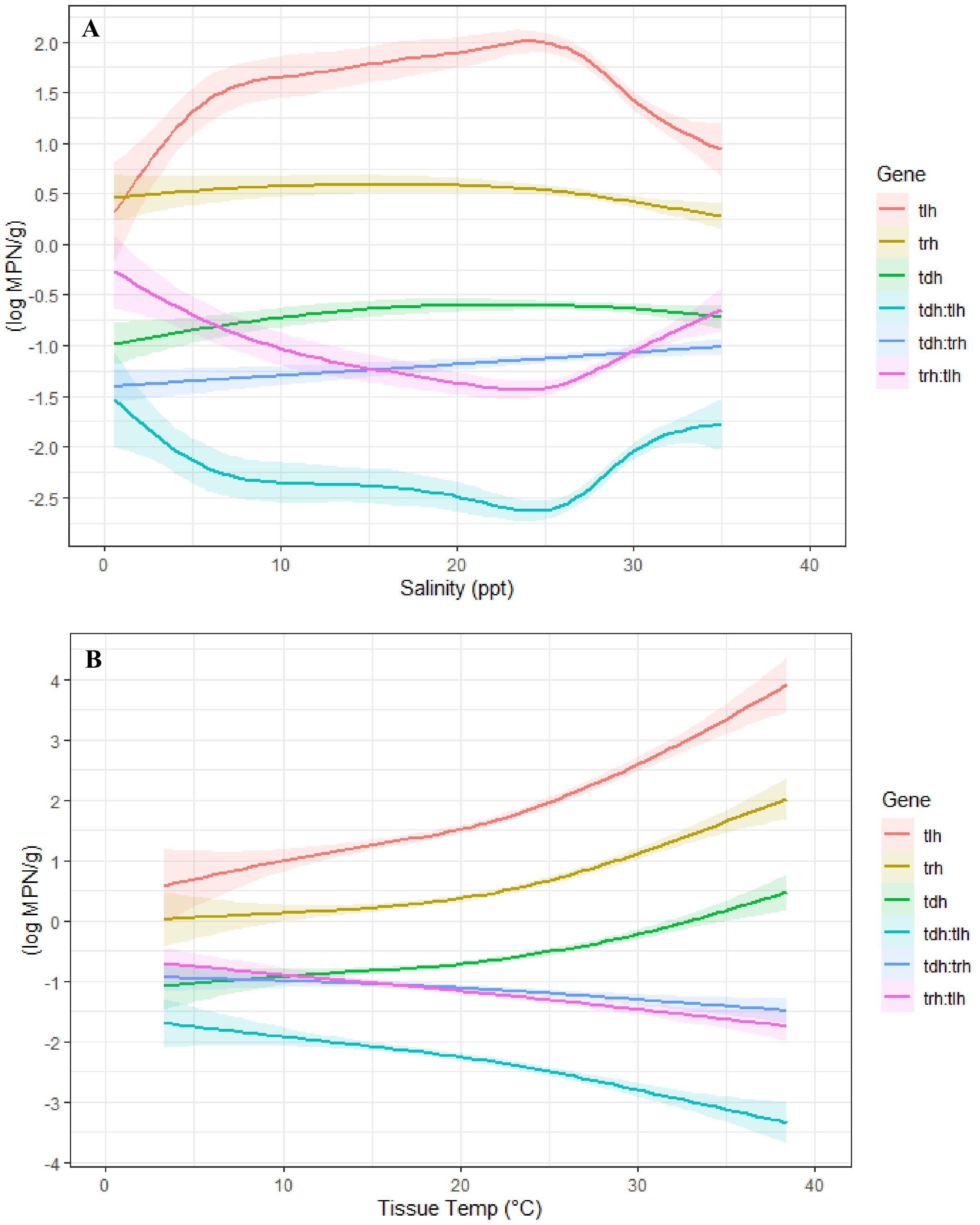

Figure S8A. Univariate lognormal regression analyses between a) salinity and b) tissue temperature, and genetic markers (representative imputation).

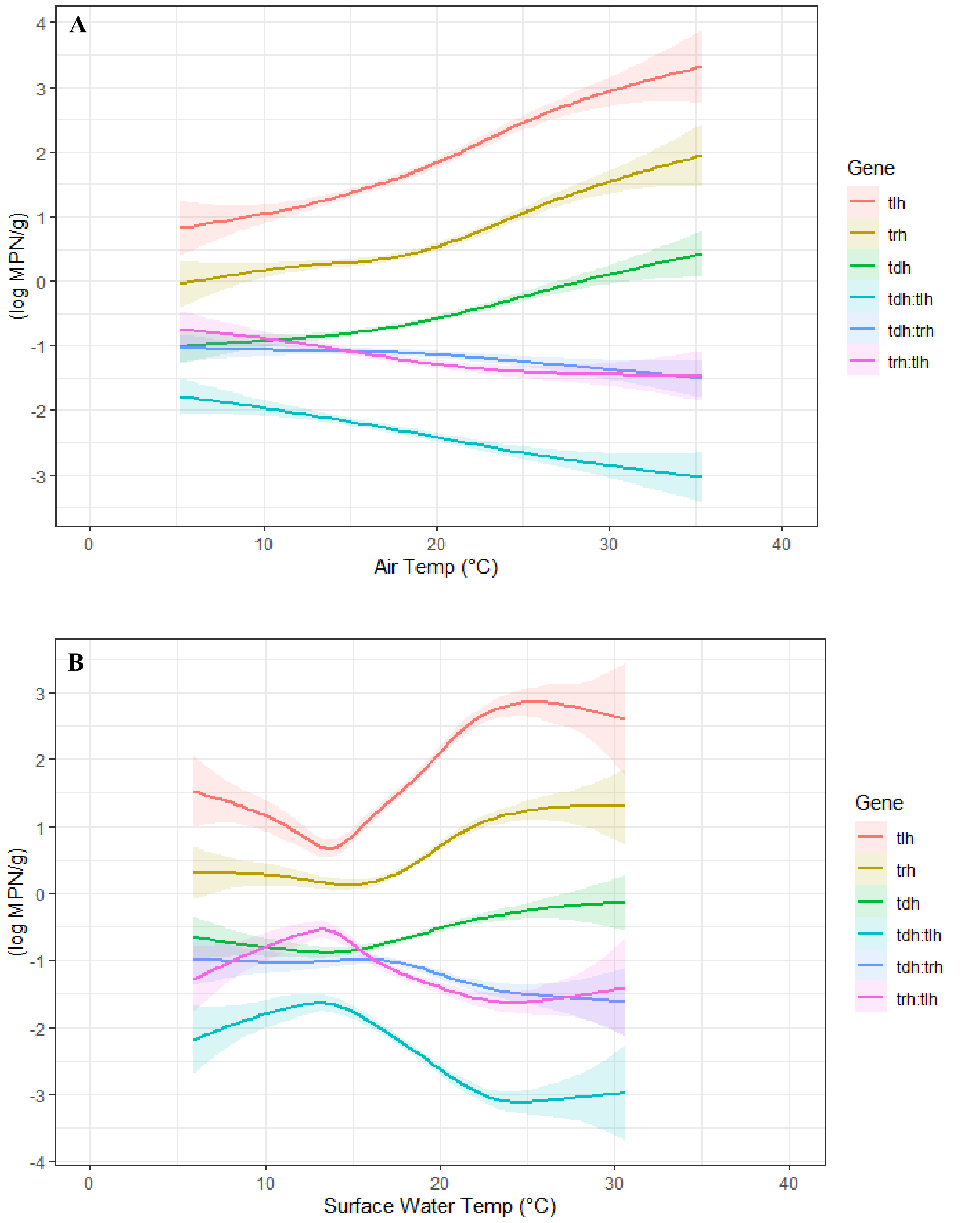

Figure S8B. Univariate lognormal regression analyses between a) ambient air temperature and b) surface water temperature, and genetic markers (representative imputation).

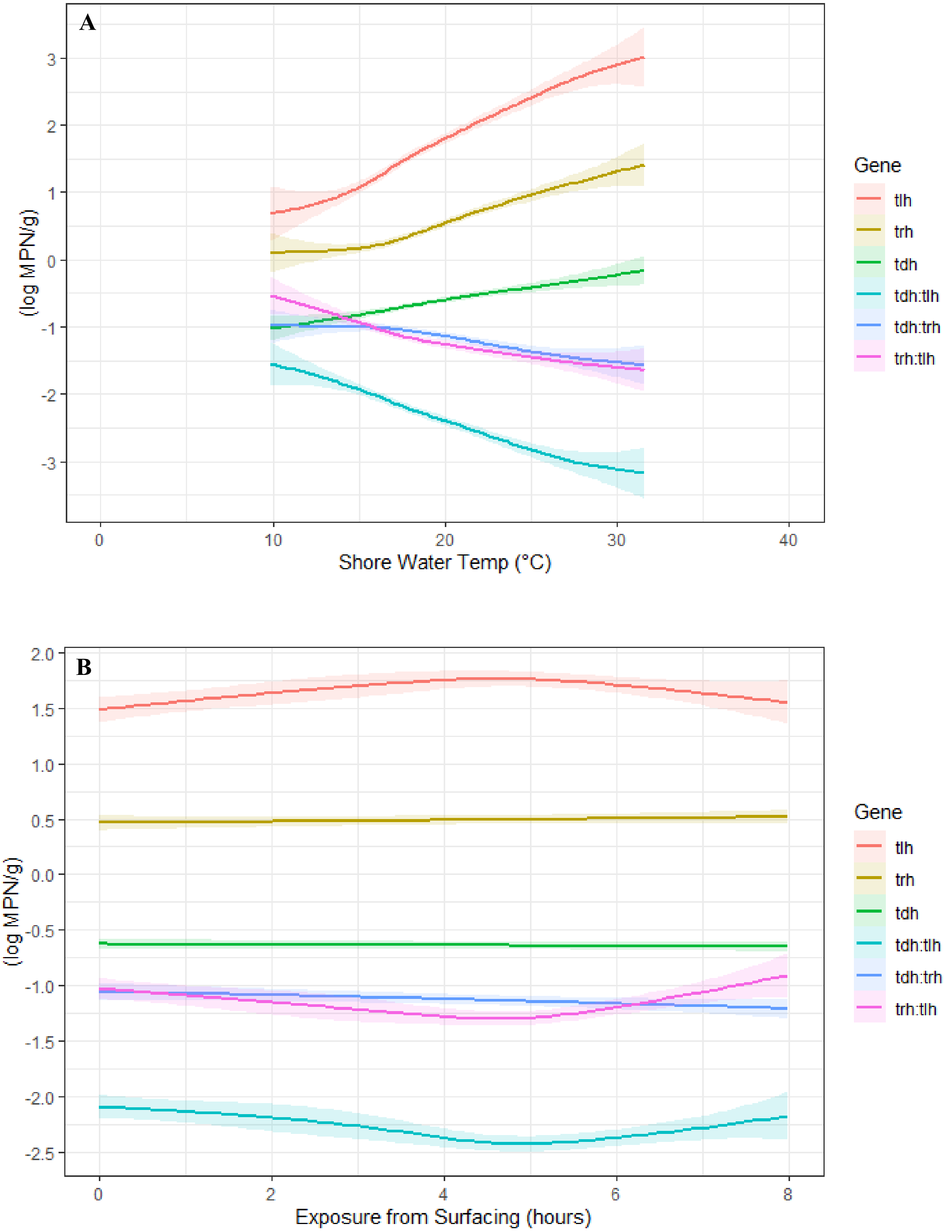

Figure S8C. Univariate lognormal regression analyses between a) shore water temperature and b) exposure from surfacing time, and genetic markers (representative imputation).

Figure S9. ACF and PACF of model residuals before and after applying nested ARMA structure to models

tlh model residual correlation **before** ARMA

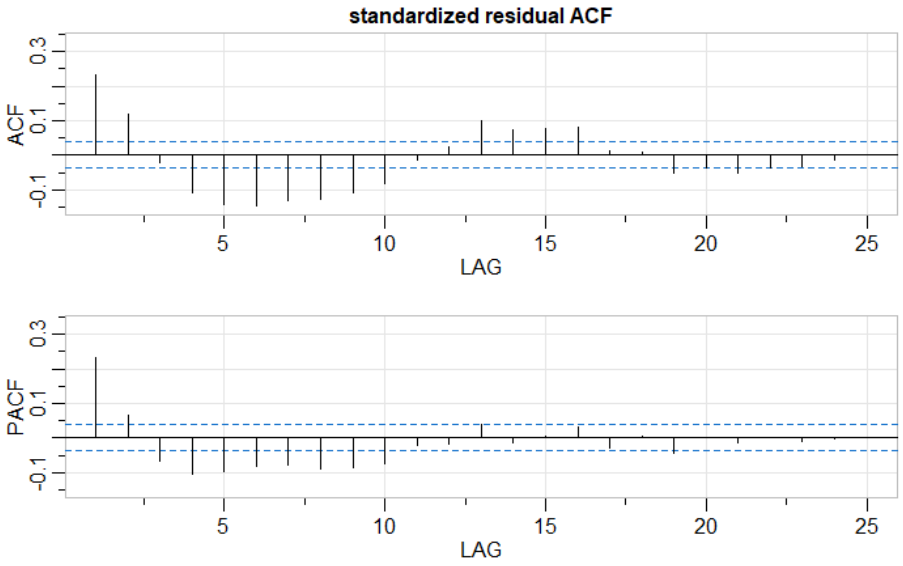

tlh model residual correlation **after** ARMA structure

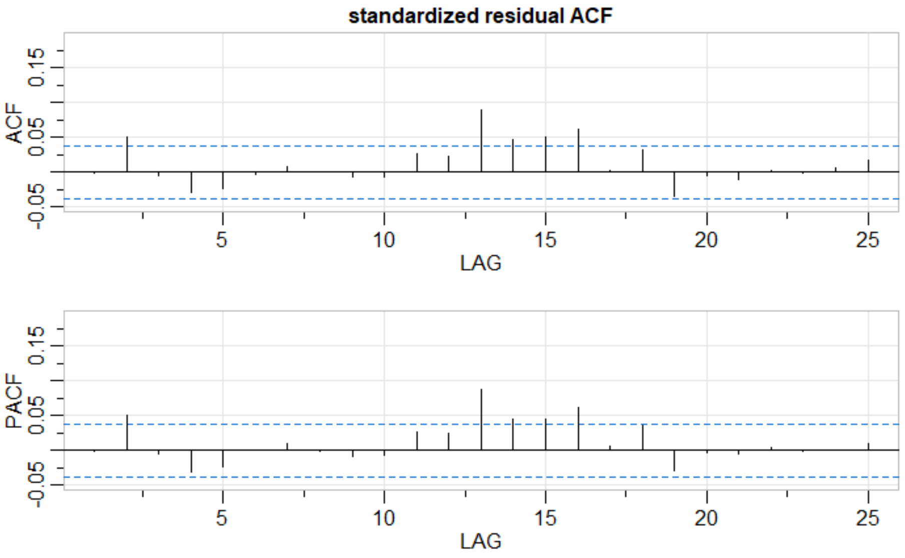

trh model residual correlation **before** ARMA structure

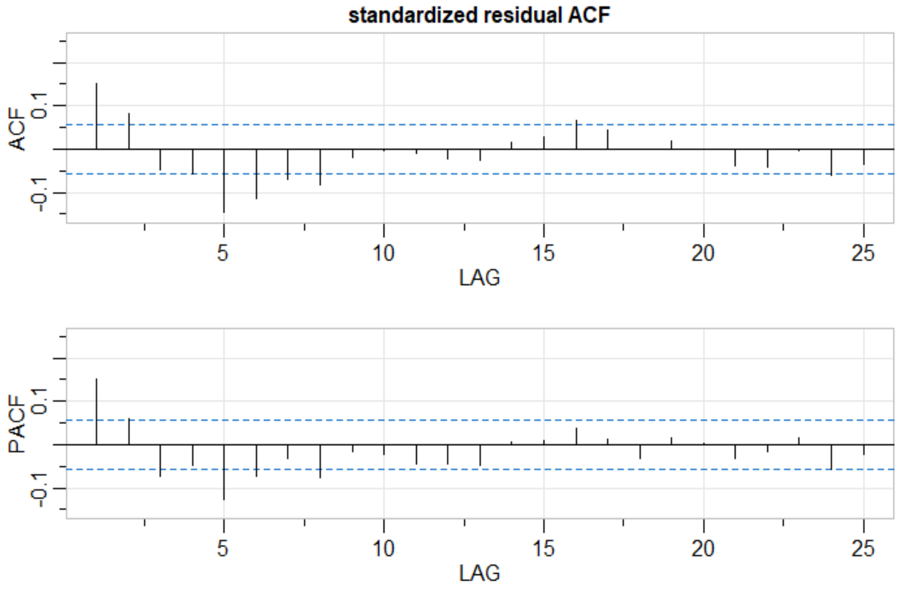

trh model residual correlation **after** ARMA structure

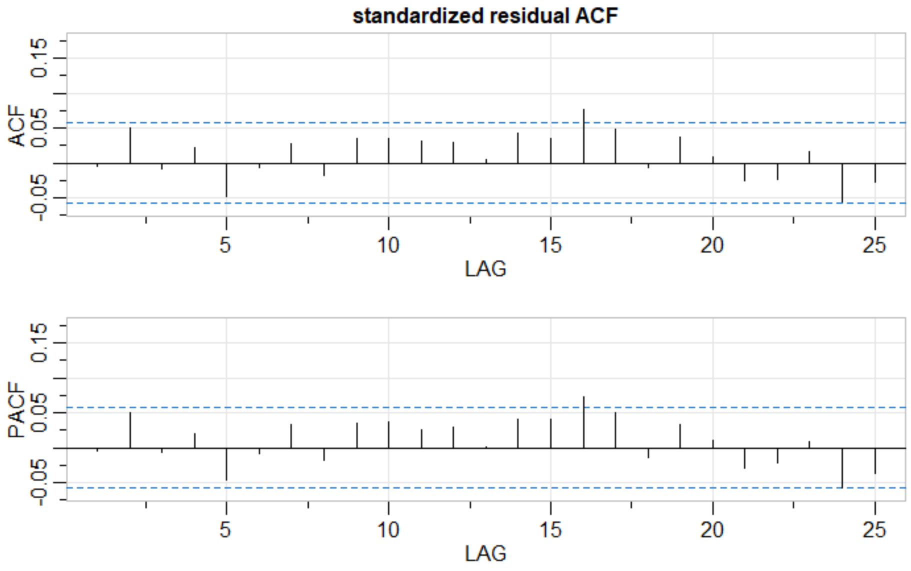

tdh model residual correlation **before** ARMA structure

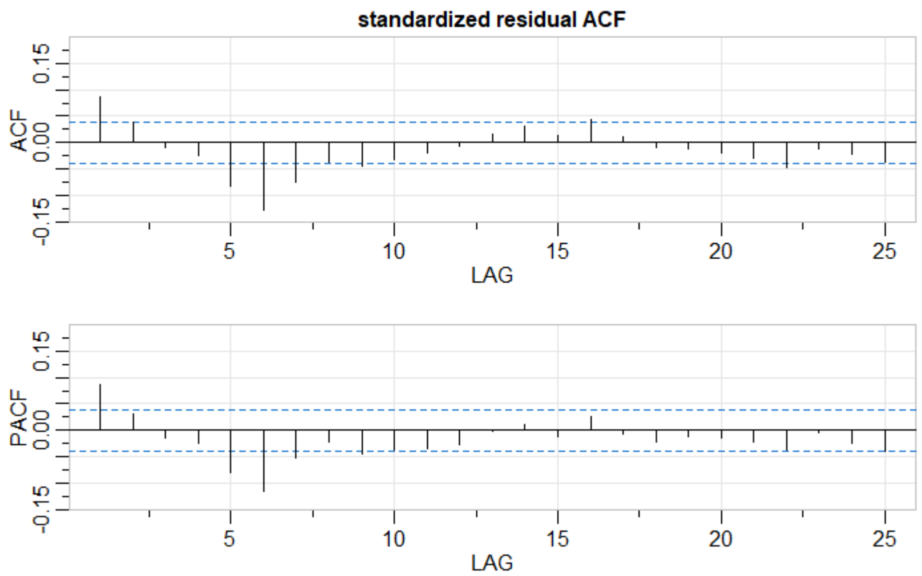

tdh model residual correlation **after** ARMA structure

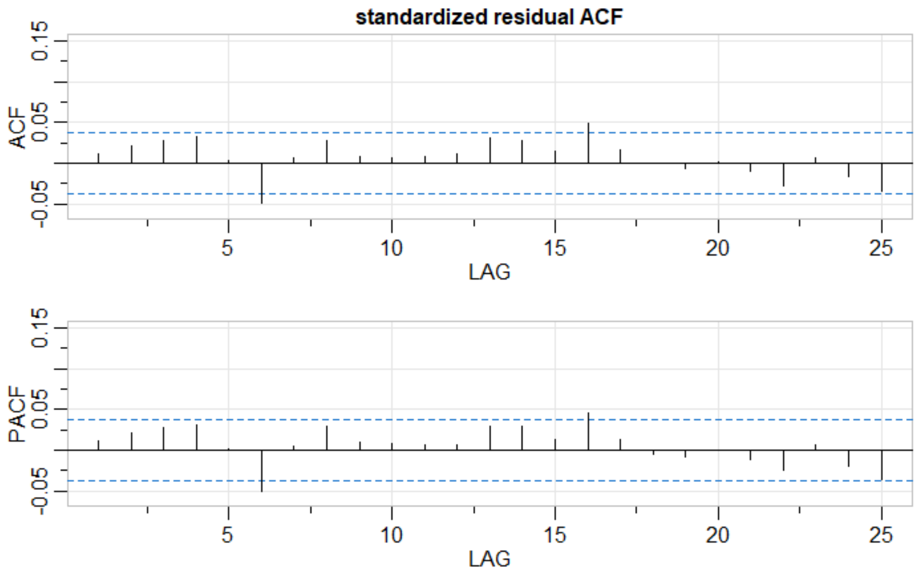

tdh:*tlh* model residual correlation **before** ARMA structure

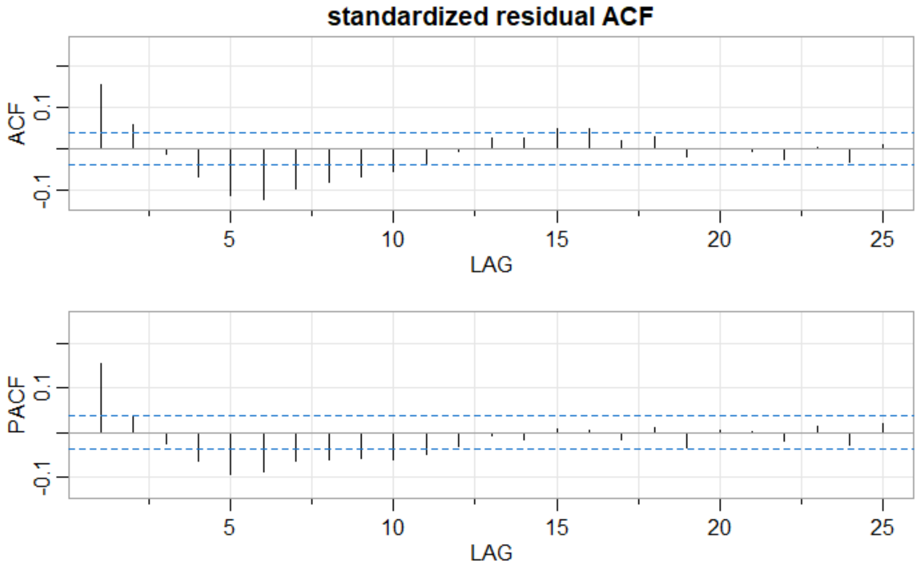

tdh:*tlh* model residual correlation **after** ARMA structure

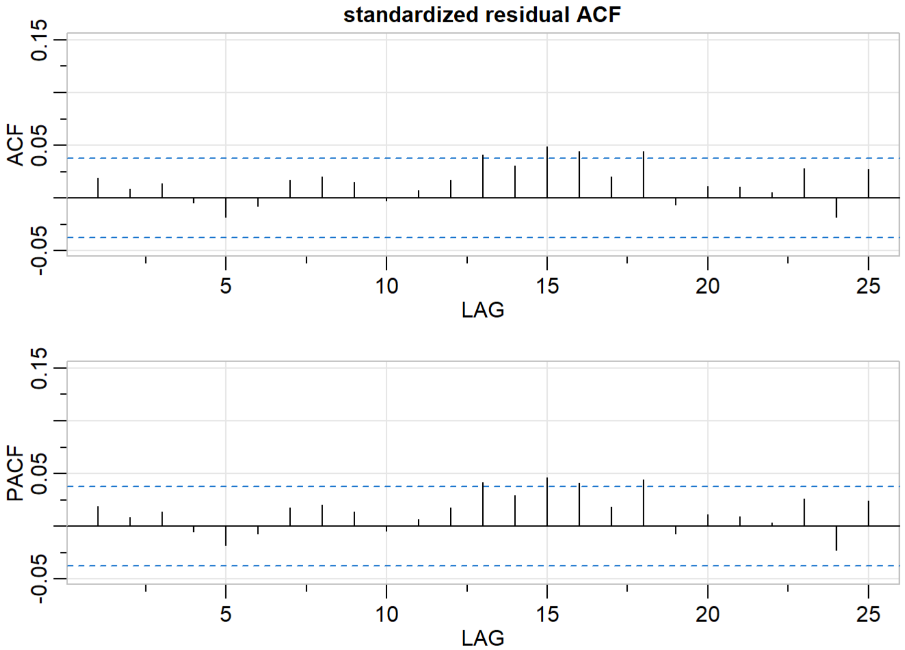

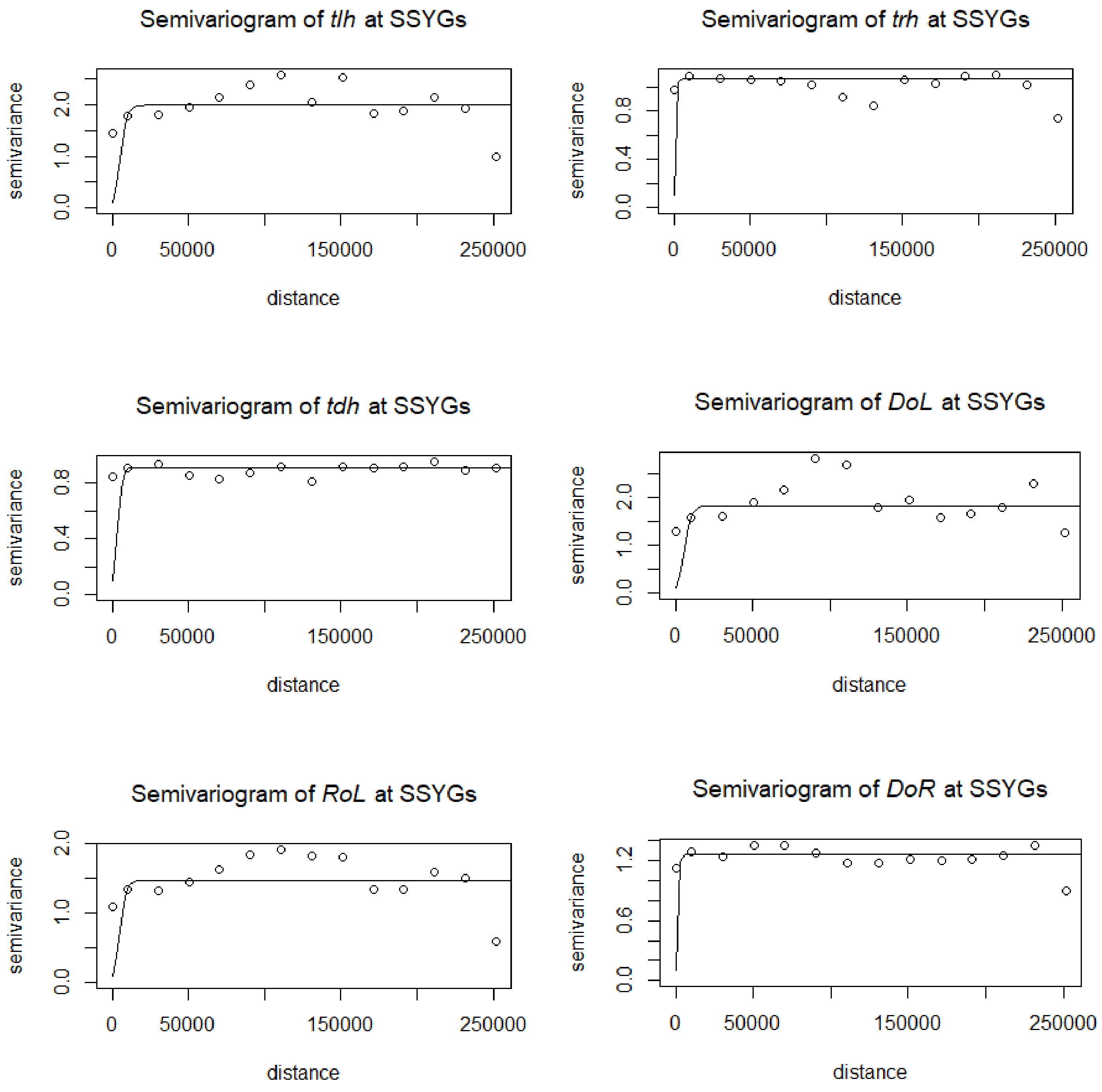

Figure S10. Variograms of spatial autocorrelation of environmental characteristics and genetic markers from model using non-Euclidian water distance function.
